# Challenges in Pediatric Cardiac Anesthesia in Developing Countries

**DOI:** 10.3389/fped.2018.00254

**Published:** 2018-10-29

**Authors:** Mirjana Cvetkovic

**Affiliations:** Depertment of Critical Care, Theatres, Anaesthesia, Pain and Sleep, Glenfield Hospital, University Hospitals of Leicester NHS Trust, Leicester, United Kingdom

**Keywords:** pediatric, cardiac, humanitarian, anesthesia, developing, mortality, education

## Abstract

**Introduction:** Approximately 90% of a million children worldwide born with congenital heart defect do not have an access to adequate pediatric cardiac care. The World Society for Pediatric and Congenital Heart Surgery, established in 2006 shifted the focus from providing individual pediatric cardiac care to developing global standards for the practice of pediatric cardiac surgery and professional education of the local teams.

**Materials and Methods:** After recognizing the challenges of the local team regarding providing safe anesthesia and functioning as a broader team, we have focused our education on simplifying anesthetic procedures and advancing structured team approach. The appropriate selection of patients and simplifying anesthetic technique should be the standard of care. We introduced structured approach to daily education using just in time teaching, case based discussions and simple skill training simulation sessions. Furthermore, we enhanced team-training approach applying tools such as WHO surgical safety checklist and implementation manual, SAFE communication, introducing KDD with SMART aim, SCAMPs, advanced protocols of care and culture change tools.

**Results:** Following a significant number of short missions to developing centers we have, within NGO, succeeded to support building and maintaining several local pediatric cardiac centers with structured approach to anesthesia and team building.

**Conclusion:** The appropriate selection of patients is one of the most important contributing factors for decreasing morbidity and mortality rate in pediatric cardiac surgery patients. The anesthesia technique for pediatric cardiac procedures should be aimed at fast-track surgery, with early extubation as a goal. Regional blocks such as paravertebral and caudal should be considered for perioperative pain control. By introducing structured approach to daily education and by enhancing team-training approach we have contributed evolving sustainable pediatric cardiac centers in developing countries.

## Introduction

It is striking that ~90% of a million children worldwide born with congenital heart defect (CHD) do not have an access to adequate pediatric cardiac care (1). Incidence of CHD ranges from 5 to 14 cases per 1,000 live births with higher absolute number in developing countries (2–4). Acquired heart diseases as rheumatic heart disease, endomyocardial fibrosis, Chagas, and Kawasaki disease are common in children in developing countries and frequently lead to premature death as a result of suboptimal medical care (1).

According to World Health Organization (WHO) a populations of two million people, requires a pediatric cardiac center performing 300–500 operations annually. That is not always the case in developing countries where, in specific areas, populations between 15 and 70 million are without a single pediatric cardiac center (5). In Asia, there is approximately one pediatric cardiac center for population of 16 million. The distribution is even less in Africa where one pediatric cardiac center covers population of 33 million (6).

Various non-governmental humanitarian organizations (NGOs) have been providing pediatric cardiac surgeries in developing countries for many years. Majority of them were short-term missions called “surgical safaris” (7). The World Society for Pediatric and Congenital Heart Surgery established in 2006 shifted the focus from providing individual pediatric cardiac care to developing global standards for the practice of pediatric cardiac surgery and professional education of the local teams (5). Furthermore, the Lancet Commission on Global Surgery published in 2015 stated that all people should have access to safe, high-quality surgical and anesthesia care. The purpose of The Lancet Commission on Global Surgery is to make this vision a reality for provision of quality surgical and anesthesia care for all (8).

## Materials and methods

### Establishing sustainable pediatric cardiac centers

#### A journey of a thousand miles begins with a single step

##### Chinese philosopher laozi (circa 604 BCE - circa 531 BCE)

Many anesthesiologists join NGOs in various missions to developing countries and function as a part of clinical, teaching, and research projects. Participating in NGO expeditions to Africa and Asia within pediatric cardiac team we have previously been exposed not only to the challenge of providing safe pediatric cardiac surgery but similarly to the challenge of providing safe general anesthesia to pediatric cardiac patients. Consequently, our NGO has identified existing local pediatric cardiac centers with potential for growing and developing into sustainable pediatric cardiac centers. Currently, the primary aim of our team is no longer to provide pediatric cardiac care. Our primary aim is focused on providing training for the local team and advancing their ability to independently diagnose and treat pediatric cardiac patients.

### Pathway

Since 2007, our NGO visited India, Malaysia, Nigeria, Kenya, Tanzania, and Mauritius. The centers visited in developing countries were carefully identified. Our teams visited centers with existing pediatric cardiac program where the basic equipment required and basic infrastructure were already in place. One team contained pediatric cardiac surgeon, surgical fellow, cardiologist, anesthesiologist, perfusionist, two intensivists, and two intensive care nurses. Our teams were visiting the local center for 1 week at a time. Continuation was provided for several months and occasionally for more than a year if required. Furthermore, our team provided a function of long-term, off-site collaborator for sustainable local centers.

### Pediatric cardiac anesthesia and team approach

**Figure 1:**
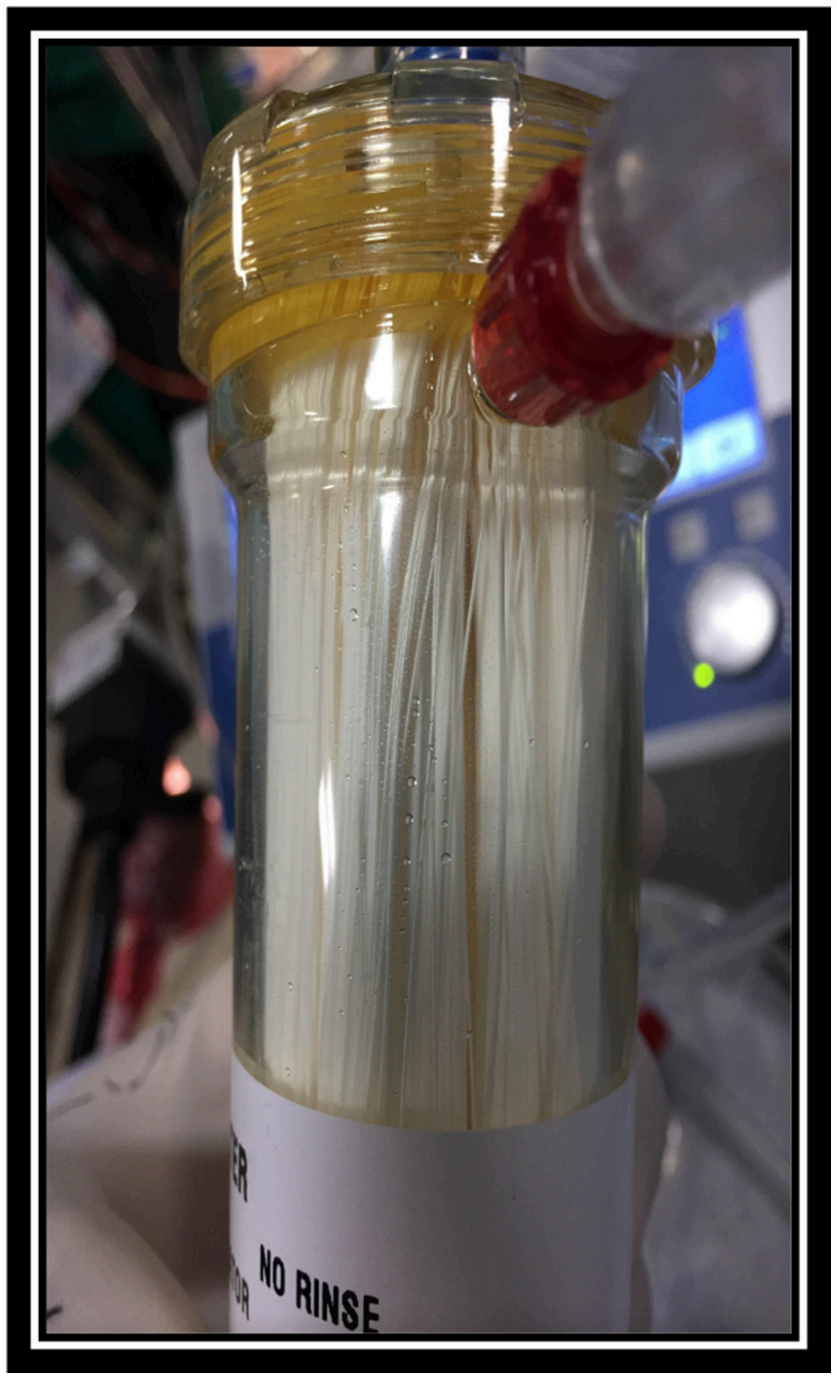
Reusing large adult oxygenator. Private collection India 2016.

**Figure 2:**
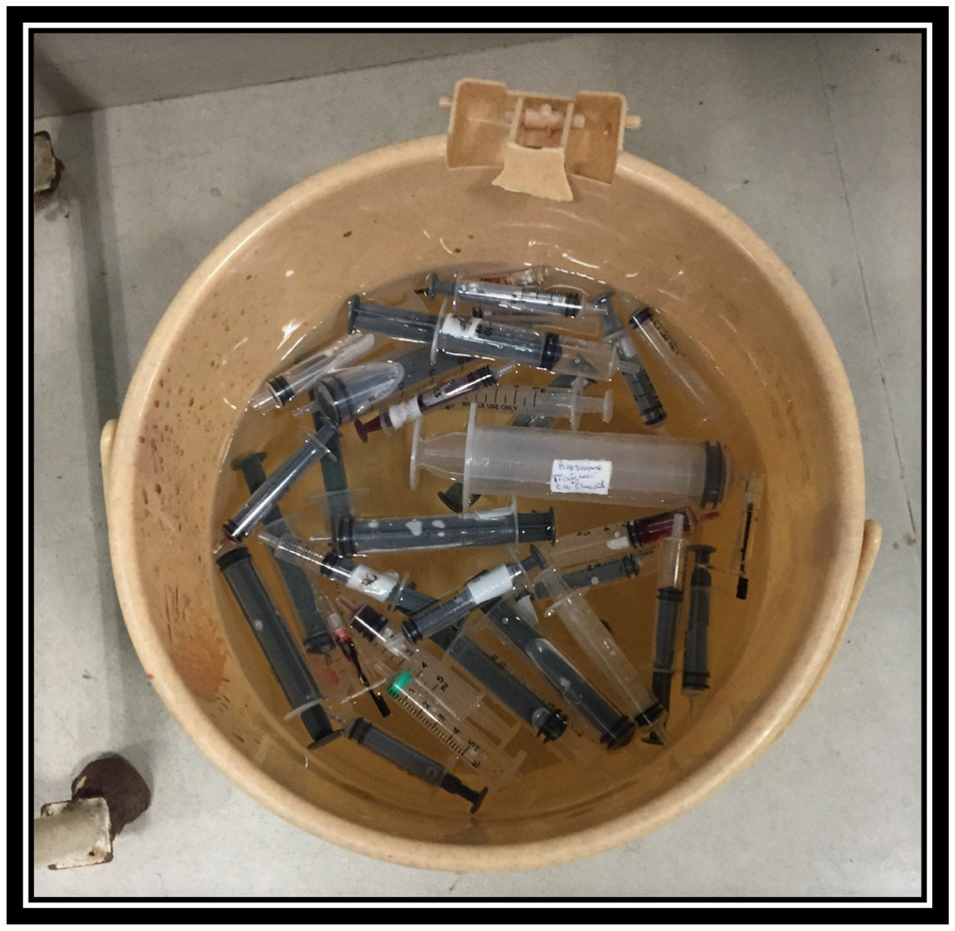
Reusing syringes. Private collection India 2.

Hence the visiting centers had basic equipment and infrastructure in place, the major challenge for the visiting anesthesiologist was not lack of equipment required. In our experience, the major challenge was lack of dedicated and sufficiently educated pediatric cardiac anesthetic team. The local anesthetic team was mainly adult trained and frequently required basic education about anatomy, physiology and appropriate anesthetic agents used for induction and maintenance of anesthesia for pediatric cardiac patients. Nevertheless, the support was required in selection of adequate endotracheal tube size (ETT), laryngoscope, intubation and ventilation techniques, ETT securing techniques, as well as selection of arterial and central line sizes, ultrasound guided insertion techniques and securing techniques. Lack of knowledge regarding cardiopulmonary bypass cannulas, circuit and oxygenator sizes (Figure 1) as well as insufficient supply of blood and blood products were often a supplementary challenge. In addition, the persistent safety treats were infection control due to reusing and recycling disposable equipment by local team (Figure 2).

Transthoracic and transoesophageal echocardiography machines were available in majority of the centers. None of the local anesthesiologists performed echocardiography. Echocardiography was performed by local cardiologists. Our team focused on improving basic pediatric cardiac anesthesiology techniques as a primary goal of our missions rather than introducing advanced echocardiography teaching for local anesthetic team.

Moreover, we have identified a second considerable challenge for local team: Functioning as a broad team of experts. Common aims, team briefs, safety checks, and structured protocol based approach to patient care were not existing.

The local centers did not have a structured method of data collection in place related to anesthetic or surgical procedures prior to our visits. Therefore, our observations were limited to descriptive rather than objective study in order to measure the impact of our implementations.

### Ways to make it better

After recognizing the challenges of the local team regarding providing safe anesthesia and functioning as a team we have focused our education on simplifying anesthetic procedures and advancing structured team approach in patient care.

### Providing safe anesthesia

#### Teaching and education

**Figure 3:**
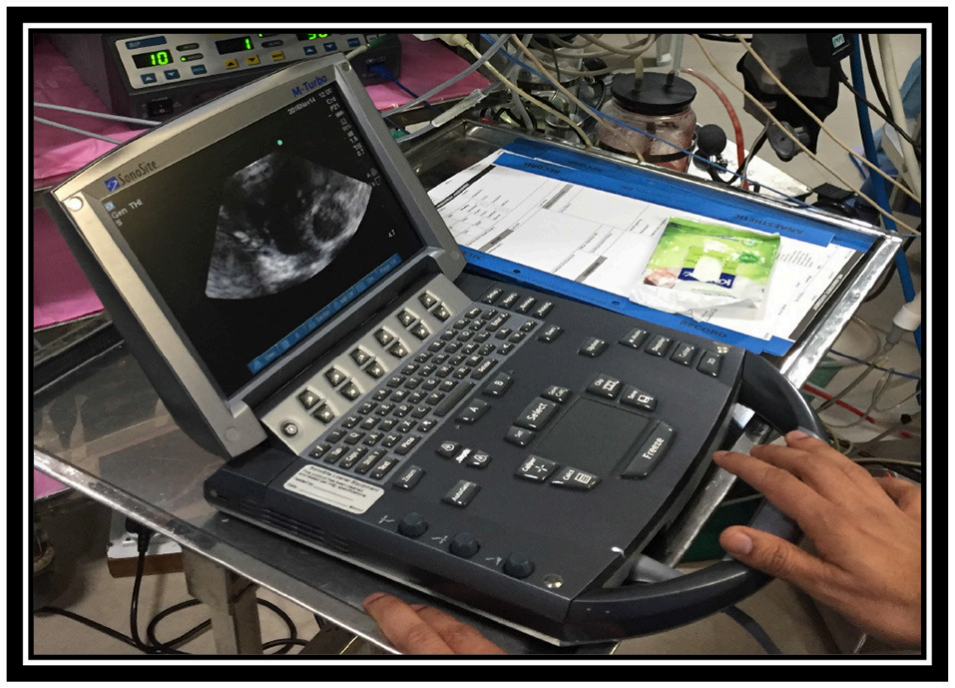
Teaching session. Private collection India 2016.

One of the goals of our team was introducing “Just in time teaching” (9). Daily education in relation to intubation and ventilation techniques, ultrasound guided line insertion (Figure 3), anesthetic agents and vasopressor support before and after cardiopulmonary bypass was provided. Weight and age related charts for ETT, laryngoscope, arterial and central line sizes as well as cardiopulmonary bypass cannulas, circuit and oxygenator sizes were introduced together with securing techniques for ETT and vascular lines.

Furthermore, we have implemented structured case based discussions and basic simulation skill training according to current anesthetic guidelines^1^. Chosen subjects of discussion reflected the majority of cases treated in the local center. Difficult airway training using difficult airway cards was conducted^2^.

In order to minimize perioperative morbidity rate, infection prevention, and control were introduced according to current standards^3^.

We provided structured and simplified approach to:

#### Case selection

**Figure 4:**
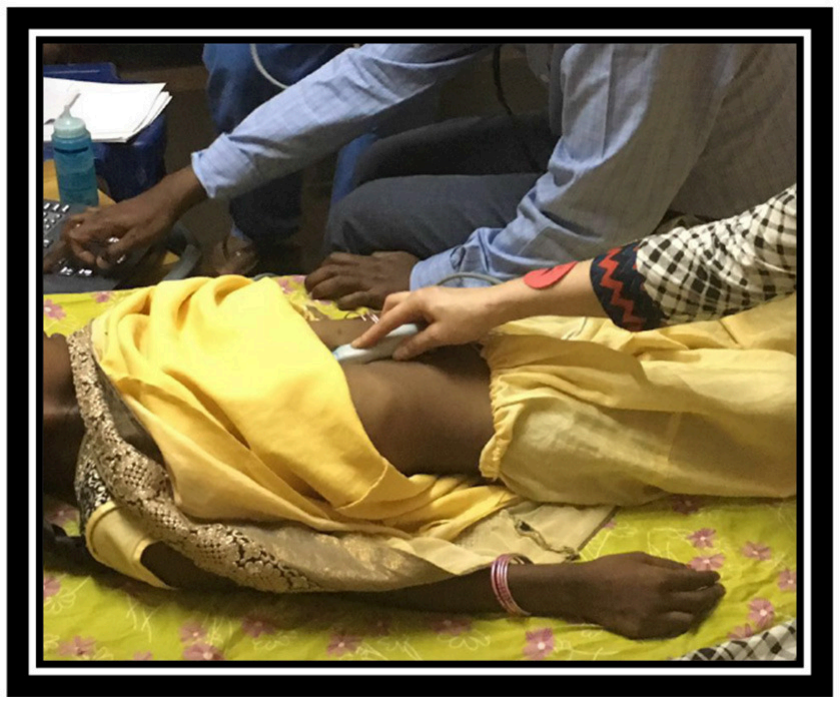
Case selection. Private collection, India 2017.

- Appropriate selection of cases including patients with simple cardiac defects (Figure 4)
- Patients with high morbidity and mortality risk or risk for complex surgical procedures should be transferred to highly specialized centers
- Procedures with a high risk of major blood loss or risk of prolonged postoperative intensive care should not be undertaken

#### Preoperative care

- Preoperative intravenous fluid resuscitation should be considered as dehydration and malnutrition were recurrent patient related issues
- Premedication should be considered
- The care providers should be using universal precautions for possible exposure to infectious diseases (HIV, hepatitis), and as infection prevention

#### Type of anesthesia

- Available appropriate anesthetic agents should be used in weight related doses
- The anesthesia technique should be aimed at fast-track surgery with early extubation in operating room (OR)
- Regional blocks such as paravertebral and caudal should be considered for perioperative pain control

Cardiology, surgical, perfusion, and intensive care training was undertaken simultaneously by other team members.

#### Structured daily team approach

In order to help the local team in maintaining the structure and competencies we have developed and introduced several standardized perioperative procedures tailored for the requirements of the local team:

- WHO surgical safety checklist and implementation manual^4^
- SAFE communication (situation awareness for everyone)^5^
- Key Driver Diagrams (KDD) with SMART (specific, measurable, achievable, relevant, time-bound) aim (10)
- SCAMPs (Standardized Clinical Assessment and Management Plan) (11)
- Culture change tools (flat organizational structure)^6^.

## Results

Supporting a pediatric cardiac center in developing countries in order to become self-sufficient and well-functioning requires time, individual enthusiasm, financial and personal investment, hard work, and dedication of NGO members. After a significant number of short missions to selected centers we have, within NGO, succeeded to support building and maintaining several local pediatric cardiac centers using structured approach to cardiology, surgery, anesthesia perfusion, and intensive care education together with team building strategy. Sustained centers have developed designated cardiology, surgical, anesthetic, perfusion, and intensive care teams and advanced team building skills. Currently, the patient care is provided on significantly higher level than prior to our visits rated by local team. Sustained centers report to have lower morbidity and mortality rate, and high success in selected surgical procedures. One center successfully provides extracorporeal membrane oxygenation (ECMO) in selected cases after collaboration with our team. Our team still functions as long-term, off-site collaborator for sustainable local centers. We are planning to provide overseas fellowships to local staff in order to advance their education and stimulate them to use the skills on return to their home country. Beyond that, we have established friendship for life.

After several years of experience our motto became the famous phrase: “The success should not be measured by the number of successful operations of any given mission, but by the successful operations that our colleagues perform after we leave” (12).

After establishing the basic care for pediatric cardiac patients we are currently aiming to establish data collection and objective measures for skill acquisition, success rate, team performance, morbidity, and mortality.

### Correspondence within teams

Team interaction within visiting and local team is very important for successful collaboration. Friendly atmosphere with zero tolerance for judgmental or discriminating behavior is fundamental for team building. Well-educated, compliant members facing challenges with professionalism are the crucial element for successful correspondence within teams. Not long ago, somebody asked me what was absolutely essential to bring on the trip. I replied, firstly your smile, and then your ultrasound equipment.

## Discussion

WHO supports the fact that “Safe surgery saves lives” (13, 14). Anesthesia is a specialty with low status in many developing countries and anesthetic services are often underdeveloped (15). It is well known that the majority of pediatric-related mortality is due to airway-related complications (16, 17). Similarly, it is a recognized fact that the number of trained pediatric cardiac anesthesiologist in developing countries is very small (18). That leads to increasing population of nonmedical anesthetic providers trained without appropriate supervision (19). A part of the anesthetic residents undertake their speciality training outside the country and frequently stay in developed countries (20). All of that contributes to two to three times increase in anesthesia related morbidity and mortality in the developing world compared with decreasing anesthesia related complications in developed countries (21–23).

Anesthesia is a technology-based specialty and relay on functioning monitoring equipment (2). Providing anesthesia in developing countries becomes highly challenging considering the fact that more than 19% of operation theaters worldwide have no pulse oxymeter (23, 24). According to millennium development program (Goal-4), oxygen supply and pulse oxymeter should be provided to every healthcare facilities especially involving pediatric patients (25). Ultrasound machine for line insertions and regional blocks is commonly not available, which increases the risk of complications furthermore. Even well-established centers have unreliable supply of basic utilities including electricity, water and oxygen (20, 26, 27), and more than 70% of developing countries lack a national blood transfusion service (1, 16). In addition, there is frequently shortage of resuscitative equipment, airway and suction devices and other intraoperative monitoring systems (24). Likewise, the increasing trend of corruption and neglect is related to the impaired healthcare systems in developing countries (25). Combination of mentioned contributing factors has a negative impact on morbidity and mortality in developing countries (24, 28). To address this concern, the main focus of visiting anesthetic team should be to reduce total perioperative and anesthetic-related mortality with evidence-based best practice. Establishing local sustainable pediatric cardiac centers in developing countries providing both initial and continued training has made the greatest impact on mortality rates in the last decade (18). It is worth remembering that adequate education of local team requires involvement of local and central government (28).

Our NGO visited existing local pediatric cardiac centers with potential for growing and developing into sustainable pediatric cardiac centers. In our experience, the major challenge of pediatric cardiac anesthesia was lack of dedicated and sufficiently educated team. The primary aim of our team was to provide training for the local team in order to advance their ability to independently diagnose and treat pediatric cardiac patients. Previous review highlights that visiting anesthesiologist frequently provides pediatric cardiac anesthesia aiming to educate local team (18). Several international Internet sites are found to be helpful tool to local team. The online tutorial of the week available on the World Federation of Societies of Anaesthesiologists (WFSA) website at http://www.anaesthesiologists.org and textbooks from the World Anesthesia Society, are useful resources of education for local team (29). Furthermore, WFSA pediatric committee offers overseas fellowships, and supports international Teach The Teachers courses (30). Overseas fellowships can provide the longer-term solution for education of the local team (31).

**Figure 5:**
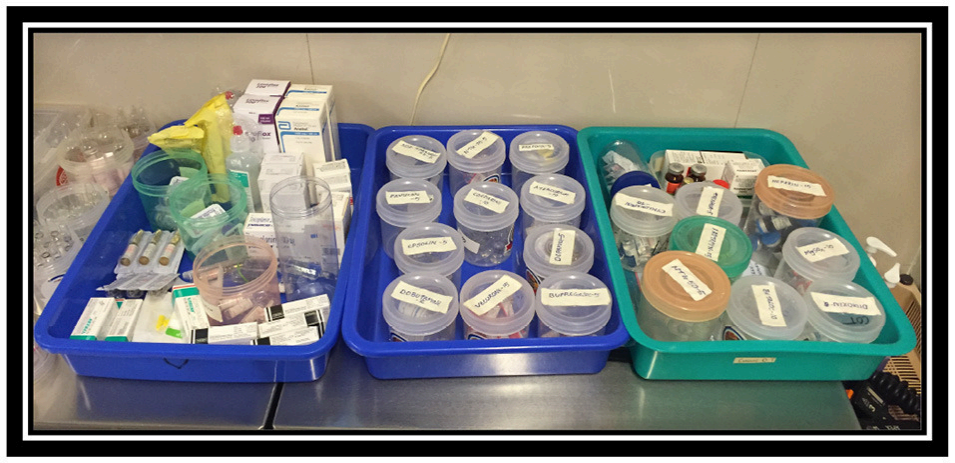
Labeling of medication. Private collection India 2016.

It is well-known that the role of simulation is highly important in skill and team training of the local team. The mannequin-based resuscitation training is found to be very effective (32, 33). Significant mortality reduction in developing countries was achieved with simulation training in new-born resuscitation (34). In general, most of the anesthesia-related cardiac events are preventable (35). Careful labeling of medications (Figure 5) and resuscitation equipment including difficult airway carts can improve patient safety. Our team has managed to introduce simple anesthetic protocols and charts for local team allowing easy interpretation and use. We have developed structured approach to daily education establishing just in time teaching, case based discussions and simple skill training simulation sessions. Furthermore, we have enhanced team-training approach applying tools such as WHO surgical safety checklist and implementation manual, SAFE communication, introducing KDD with SMART aim, SCAMPs, advanced protocols of care and culture change tools. By introducing structured approach to daily education and by enhancing team-training approach we have contributed evolving sustainable pediatric cardiac centers in developing countries.

### Limitations of the study

The local centers did not have a structured method of data collection in place related to anesthetic or surgical procedures prior to our visits. Therefore, this study is subjective and observational limited to description of methods and techniques. Currently, the impact of our implementations is rated by local team. Sustained centers report to have lower morbidity and mortality rate, and high success in selected surgical procedures.

## Conclusion

Establishing local sustainable pediatric cardiac centers in developing countries providing both initial and continued training has made the greatest impact on mortality rates in the last decade (18). It requires careful determination of adequate center with potential for growing into sustainable pediatric cardiac center. The appropriate selection of cases including patients with simple cardiac defects is one of the most important contributing factors for decreasing morbidity and mortality rate in pediatric cardiac surgery patients. Anesthesia technique is a global challenge. The main focus of visiting anesthetic team should be to reduce total perioperative and anesthetic-related mortality with evidence-based best practice. Simplification of the care should be the primary anesthetic technique for pediatric cardiac procedures, and should be aimed at fast-track surgery, with early extubation as a goal. Regional blocks such as paravertebral and caudal should be considered for perioperative pain control.

Correspondingly, team performance is a considerable challenge for local team.

By introducing structured approach to daily education using just in time teaching, case based discussions and simple skill training simulation sessions, together with enhancing team-training approach by applying tools such as WHO surgical safety checklist and implementation manual, SAFE communication, KDD with SMART aim, SCAMPs, advanced protocols of care and culture change tools we have contributed evolving sustainable pediatric cardiac centers in developing countries.

## Author contributions

The author confirms being the sole contributor of this work and has approved it for publication.

### Conflict of interest statement

The author declares that the research was conducted in the absence of any commercial or financial relationships that could be construed as a potential conflict of interest. The handling Editor declared a shared affiliation, though no other collaboration, with the author MC.

## Footnotes

^1^https://www.rcoa.ac.uk/system/files/GPAS-2018-10-PAEDIATRICS.pdf (Assessed May 17, 2018).

^2^https://www.das.uk.com/guidelines/paediatric-difficult-airway-guidelines (Assessed May 17, 2018).

^3^https://www.nice.org.uk/guidance/QS61 (Assessed May 17, 2018).

^4^http://www.who.int/patientsafety/safesurgery/ss_checklist/en/ (Assessed May 17, 2018).

^5^www.steris.com (Assessed May 17, 2018).

^6^https://bcpsqc.ca/resource/culture-change-toolbox/, Culture toolbox (Assessed May 17, 2018).
